# Etiology and Types of Seizures in Patients Presenting to a Tertiary Care Hospital in Karachi: A Cross-Sectional Study

**DOI:** 10.7759/cureus.9194

**Published:** 2020-07-15

**Authors:** Wajid Jawaid, Qamar Nisa, Sumera R Umer, Sidra J Barry, Amir Qureshi, Naila N Shahbaz

**Affiliations:** 1 Neurology, Dow University of Health Sciences, Karachi, PAK

**Keywords:** phenomenology of epilepsy, primary epilepsy, secondary epilepsy, epilepsy in pakistan, epilepsy

## Abstract

Introduction

Epilepsy is a burdensome disorder for affected individuals and community. There is limited data available on the epidemiological aspects of seizures in Pakistan and further research is necessary. We aimed to fill this gap by studying this information in epilepsy patients presenting to our neurology department. The purpose of this study is to evaluate the causes and types of seizures among the target population.

Method

This is a cross-sectional study conducted at the Department of Neurology, Dr. Ruth K.M. Pfau Civil Hospital Karachi. In this study we evaluated the causes and types of seizures among patients presenting to our department during the two-year study duration (January 2018-December 2019). Informed consent was taken. Detailed history was taken including features of seizure episodes, age at first seizure, family history and comorbid conditions. Relevant investigations were carried out. The data was compiled to deduce the relevant information using SPSS v20.0. T-test and Chi-square were used for analyzing the data.

Results

A total of 996 patients presented during the study duration. Primary seizures were found in 58% cases while secondary seizures were found in 42% cases. This distribution was more equal in children with 49.6% primary seizures and 50.4% secondary seizures; the gap widened in adults with 64.3% primary seizures and 35.7% secondary seizures. The most common cause of secondary seizures was neonatal encephalopathy which was present in 18.7% patients, followed by traumatic head injury in 18.2% patients. Central nervous system (CNS) infection was the cause in 17.9% patients, cerebral tumors in 14.1% patients, stroke in 11.5%, metabolic encephalopathy in 7.4%, febrile seizures in 6.5% and CNS malformations in 5.7% patients. The top three causes in children were neonatal encephalopathy (28.3%), CNS infections (19.3%) and febrile seizures (12.7%). Adults with secondary seizures were diagnosed most often with head trauma (25.2%), cerebral tumors (19.9%) and stroke (18.4%) as causative factors. The most common type of seizures was generalized onset tonic-clonic seizures which was found in 73.0% patients followed by focal to bilateral tonic-clonic seizures in 8.9% patients. Other types of seizures included focal aware seizures in 5.0%, mixed seizure types in 4.2%, focal impaired awareness seizures in 3.1%, absence seizures in 2.7%, myoclonic seizures in 2.0% and atonic seizures in 1.0% patients. Seizures in children were mostly generalized onset tonic-clonic seizures (75.4%), mixed seizure types (5.7%) and focal to bilateral tonic-clonic seizures (5.2%). In adults the three most common types corresponded to the overall result: generalized onset tonic-clonic seizures (71.2%), focal to bilateral tonic-clonic seizures (11.6%) and focal aware seizures (6.6%).

Conclusion

We found that the most common cause of seizures overall in our study population was primary seizures, though primary and secondary seizures were more evenly present in children. Among secondary causes neonatal encephalopathy stood out as the most common cause in children; head trauma was the predominant cause in adults. Most common type of seizures overall and in adults was generalized onset tonic-clonic seizures, followed by focal to bilateral tonic-clonic and focal aware seizure types. Pediatric patients presented most often with generalized onset tonic-clonic seizures, followed by mixed seizure types and focal to bilateral tonic-clonic seizures.

## Introduction

Approximately 10% of the world’s population experience seizures at least once in life [1]. Seizures need appropriate investigations to find the underlying cause. Individuals who have two or more unprovoked seizures are diagnosed with epilepsy which is one of the most common neurological disorders. It affects around 70 million people worldwide [2]. Pakistan had 1.38 million cases according to Aziz et al. in 1994. They found the prevalence of epilepsy in Pakistan as 9.99 per 1000 people [3]. There is scarce data available on the epidemiological aspects of seizures and other neurological conditions in Pakistan and further research is badly needed. We aimed to fill this gap by studying this information in all epilepsy patients presenting to neurology department, over a period of two years, in Dr. Ruth K.M. Pfau Civil Hospital Karachi.

The causes of seizures are diverse. They can be primary, or secondary with vascular, infective, metabolic, neoplastic, degenerative and genetic causes. Head injuries can also cause seizures either immediately or many years later. In the elderly, stroke and neurodegenerative conditions frequently lead to seizures [4].

The aim of this study is to evaluate the causes and types of seizures among patients presenting to a tertiary care hospital in Karachi. Current data in this regard will help the policy makers in ensuring availability of the best drugs based on seizure types and causes. By identifying the most common causes of epilepsy, this study also intends to create opportunity and awareness for preventing these causes.

## Materials and methods

This is a cross-sectional study conducted at the Department of Neurology, Dr. Ruth K.M. Pfau Civil Hospital Karachi. In this study we evaluated the causes of seizures among patients presenting to our department during the two-year study duration (January 2018 to December 2019). The study was approved by the institutional review board. Trained doctors were asked to interview the patients after taking informed consent. Demographic variables such as age, gender and socio-economic status were recorded. Detailed history was taken including features of seizure episodes, age at first seizure, family history and co-morbid conditions. Complete physical examination was performed. Baseline investigations were ordered such as complete blood picture, liver function tests, renal function tests, serum electrolytes, serum urea and creatinine levels. Causes of seizures were evaluated using detailed history and diagnostic modalities such as electroencephalogram (EEG), magnetic resonance imaging (MRI) brain, computed tomography (CT) brain scan, and cerebrospinal fluid (CSF) sampling. MRI was performed in majority of patients while CT scan was done in only a few patients in whom MRI was not necessary for diagnosis. CSF studies were reserved for those patients in whom infective or inflammatory causes were suspected. Seizures were diagnosed and classified based on the criteria defined by International League Against Epilepsy commission on classification and terminology of seizures [5]. Patients of any age with generalized onset tonic-clonic, focal aware, focal impaired awareness, focal to bilateral tonic-clonic, myoclonic, atonic or absence seizures were included in the study. Patients with conversion disorder or psychogenic non-epileptic seizures were not included in the study. Data was analyzed using SPSS v20.0 (IBM Corp., Armonk, NY). T-test and Chi-square were used for analysis. P-values less than 0.05 were considered significant.

## Results

**Figure 1:**
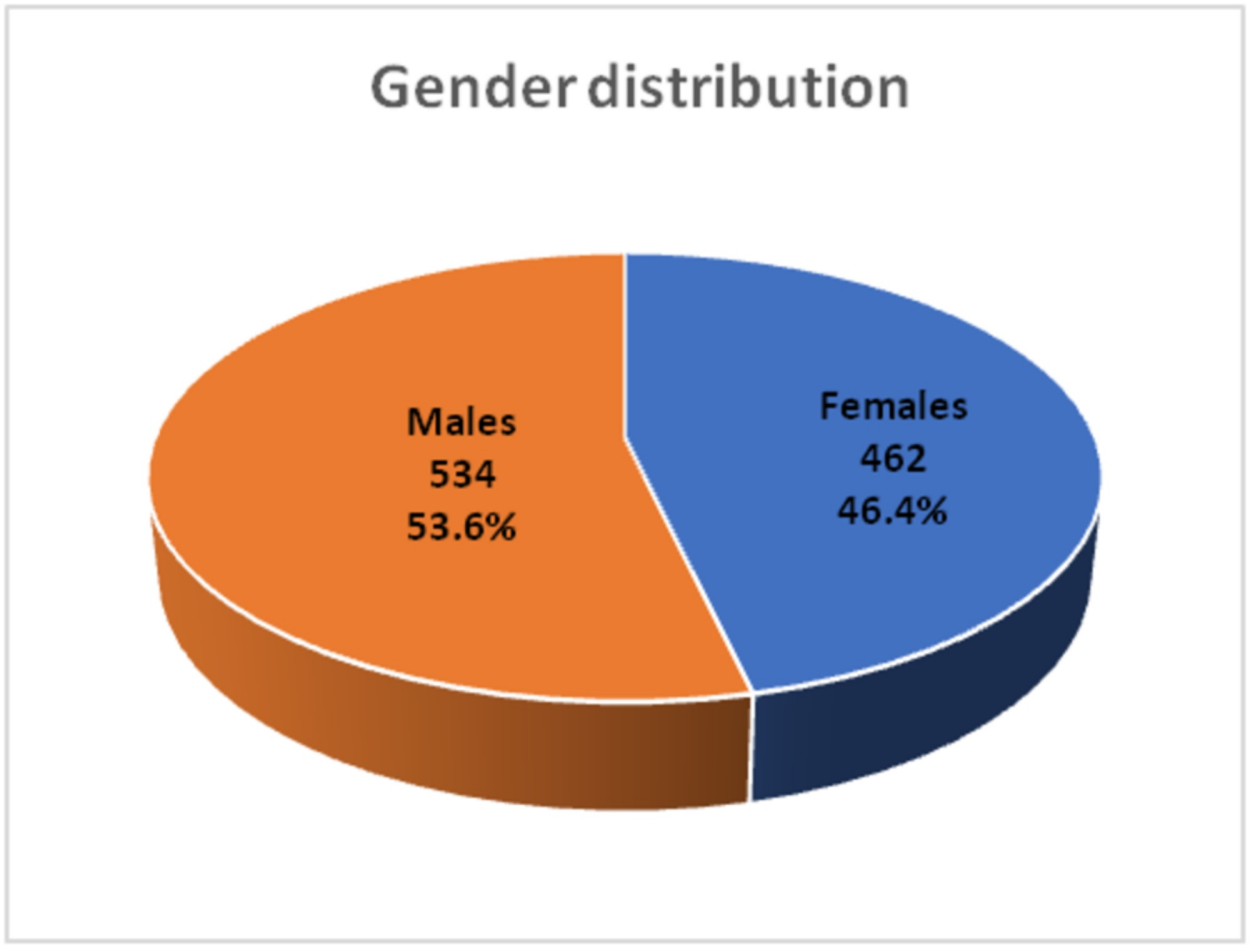
Gender Distribution

A total of 996 patients presented during the study duration. This makes 10.5% of the total number of 9444 patients who presented to neurology department in this period. The mean age of our patients was 21.4 ± 3.2 years. Among them 534 (53.6%) were males and 462 (46.4%) were females (Figure 1).

**Figure 2:**
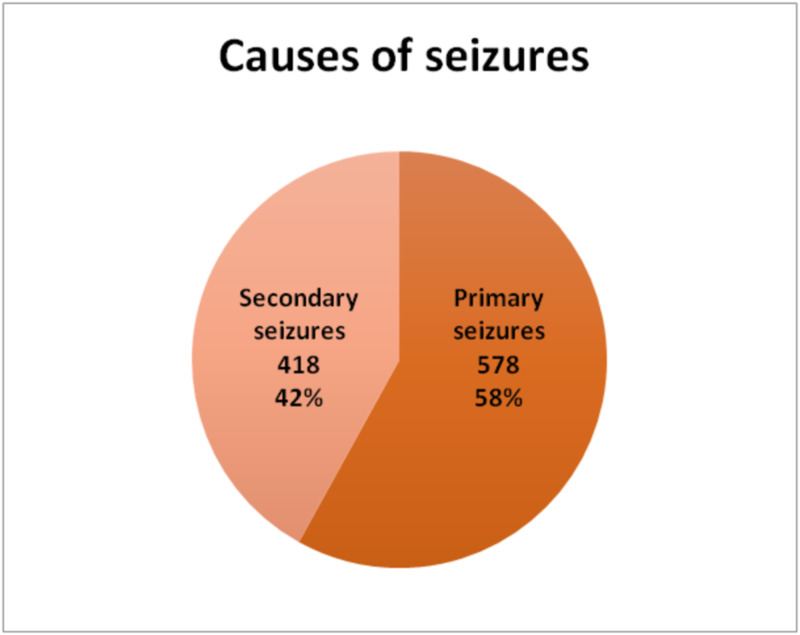
Causes of Seizures in All Patients

**Figure 3:**
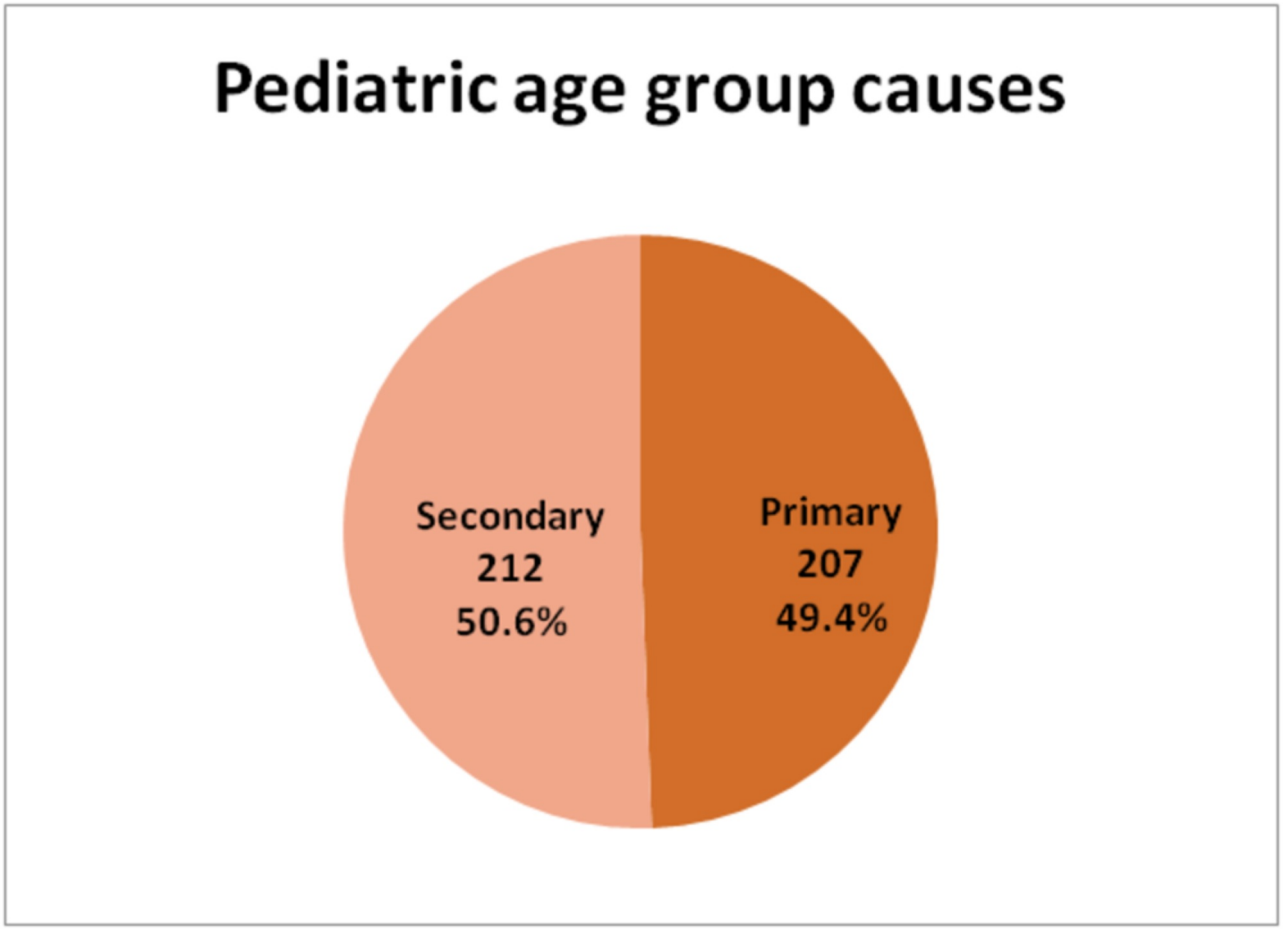
Causes of Seizures in Pediatric Patients

**Figure 4:**
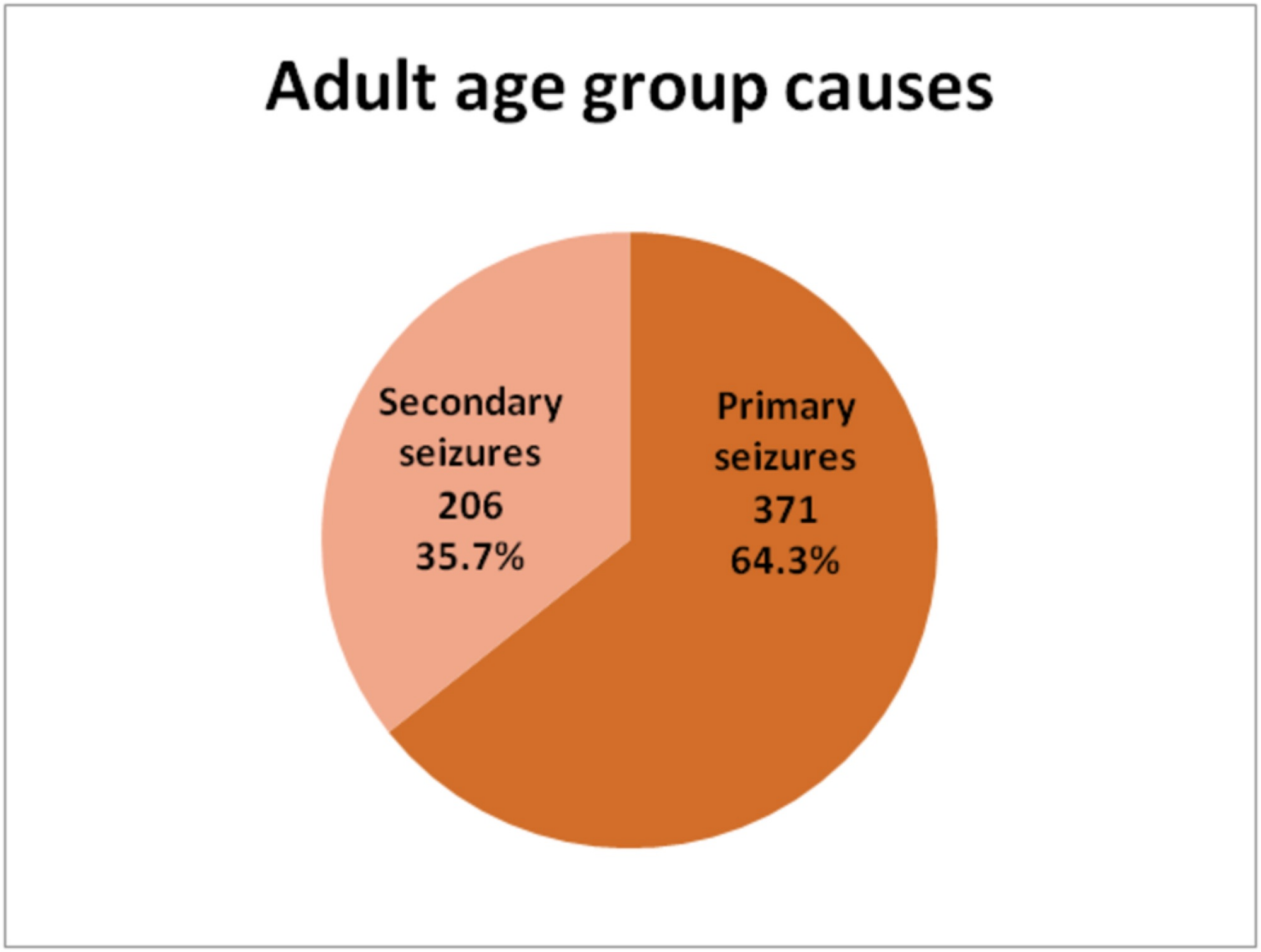
Causes of Seizures in Adult Patients

Primary seizures were found in 578 (58%) of total cases while secondary seizures were found in 418 (42%) cases. Among pediatric patients primary and secondary seizures were more evenly distributed - primary seizures 207 (49.6%) and secondary seizures 212 (50.4%). Adult patients with primary seizures were 371 (64.3%) and 206 (35.7%) adults presented with secondary seizures (Figures 2-4).

The age distribution is shown in Table 1.

**Table 1:** Age Distribution

AGE GROUP	NUMBER OF PATIENTS
Pediatric (less than 18 years)	419
Adult (18 years or more)	577
AGE RANGE	NUMBER OF PATIENTS
0 to 5 years	115
6 to 10 years	67
11 to 15 years	87
16 to 20 years	191
21 to 25 years	121
26 to 30 years	97
31 to 35 years	41
36 to 40 years	22
41 to 45 years	42
46 to 50 years	45
51 to 55 years	62
56 to 60 years	52
More than 60 years	54
TOTAL NUMBER OF PATIENTS	996

**Figure 5:**
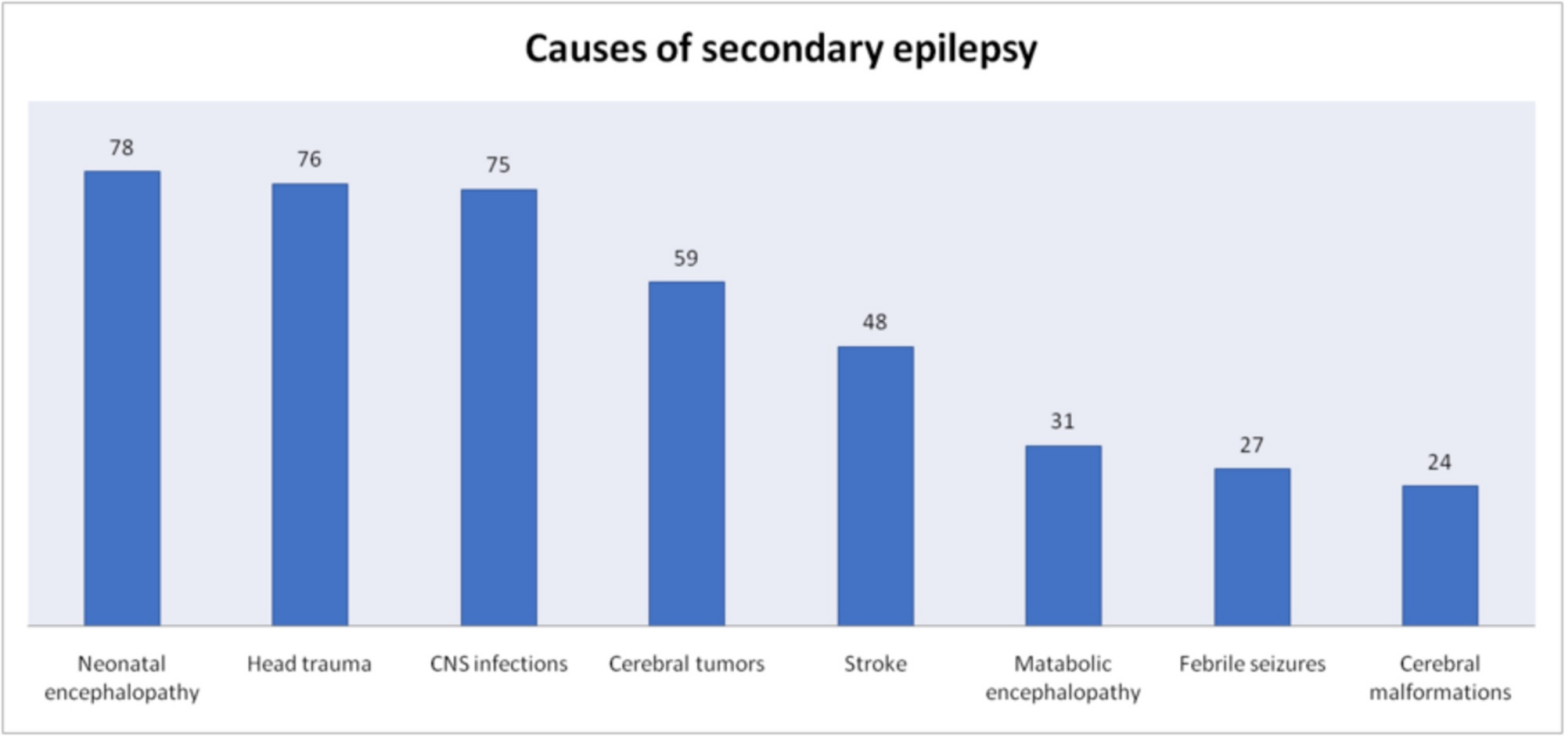
Causes of Secondary Seizures in All Patients

**Figure 6:**
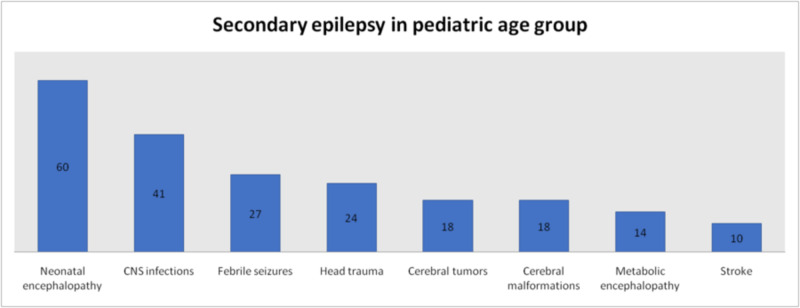
Causes of Secondary Seizures in Pediatric Patients

**Figure 7:**
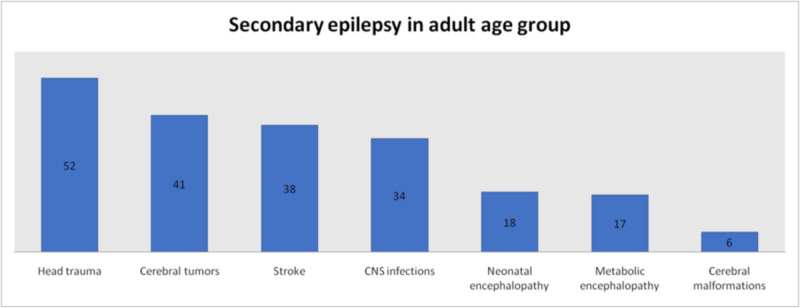
Causes of Secondary Seizures in Adult Patients

Overall the most common cause of secondary seizures was neonatal encephalopathy which was the cause in 18.7% (n = 78) patients, followed by traumatic head injury in 18.2% (n = 76) patients. CNS infection was the cause in 17.9% (n = 75) patients, cerebral tumors in 14.1% (n = 59) patients, stroke in 11.5% (n = 48), metabolic encephalopathy in 7.4% (n = 31), febrile seizures in 6.5% (n = 27) and cerebral malformations in 5.6% (n = 24) patients. The foremost cause of secondary seizures in pediatric population was neonatal encephalopathy which was found in 28.3% (n = 60), followed by CNS infections in 19.3% (n = 41). Febrile seizure was the cause in 12.7% (n = 27), head trauma in 11.3% (n = 24), cerebral tumors in 8.5% (n = 18), cerebral malformations in 8.5% (n = 18), metabolic encephalopathy in 6.6% (n = 14) and stroke in 4.7% (n = 10). Most prominent among the causes of secondary seizures in adults was head trauma which was found in 25.2% (n = 52), followed by cerebral tumors in 19.9% (n = 41) patients. Other causes were stroke in 18.4% (n = 38), CNS infections in 16.5% (n = 34), neonatal encephalopathy in 8.7% (n = 18), metabolic encephalopathy in 8.2% (n = 17) and cerebral malformations in 2.9% (n = 6) patients (Figures 5-7).

The most common type of seizures overall was generalized onset tonic-clonic seizures which was found in 73.0% (n = 727) patients, followed by focal to bilateral tonic-clonic seizures in 8.9% (n = 89) patients. Other types of seizures found in our study population included focal aware seizures in 5% (n = 50), mixed seizure types in 4.2% (n = 42), focal impaired awareness seizures in 3% (n = 31), absence seizures in 2.7% (n = 27), myoclonic seizures in 2% (n = 20) and atonic seizures in 1% (n = 10) patients. Among the pediatric population most common type of seizures was generalized onset tonic-clonic seizures in 75.4% (n = 316), followed by mixed seizure types in 5.7% (n = 24). Other seizure types in pediatric patients were focal to bilateral tonic-clonic in 5.2% (n = 22), absence seizures in 4.8% (n = 20), focal aware seizures in 2.9% (n = 12), myoclonic seizures in 2.9% (n = 12), atonic seizures in 1.9% (n = 8) and focal aware seizures in 1.2% (n = 5). Among the adult patients most common type of seizures was generalized onset tonic-clonic seizures in 71.2% (n = 411), followed by focal to bilateral tonic-clonic seizures 11.6% (n = 67). Other seizure types in adults were focal aware seizures in 6.6% (n = 38), focal impaired awareness seizures in 4.5% (n = 26), mixed seizure types in 3.1% (n = 18), myoclonic seizures in 1.4% (n = 8), absence seizures in 1.2% (n = 7) and atonic seizures in 0.3% (n = 2) (Tables 2-4).

**Table 2:** Types of Seizures in All Patients

TOTAL NUMBER OF PATIENTS	N = 996
Generalized onset tonic-clonic seizures	n = 727 (73.0%)
Focal aware seizures	n = 50 (5.0%)
Focal impaired awareness seizures	n = 31 (3.1%)
Focal to bilateral tonic-clonic seizures	n = 89 (8.9%)
Absence seizures	n = 27 (2.7%)
Myoclonic seizures	n = 20 (2.0%)
Atonic seizures	n = 10 (1.0%)
Mixed seizure types	n = 42(4.2%)

**Table 3:** Types of Seizures in Pediatric Patients

TOTAL NUMBER OF PEDIATRIC PATIENTS	N = 419
Generalized onset tonic-clonic seizures	n = 316 (75.4%)
Focal aware seizures	n = 12 (2.9%)
Focal impaired awareness seizures	n = 5 (1.2%)
Focal to bilateral tonic-clonic seizures	n = 22 (5.2%)
Absence seizures	n = 20 (4.8%)
Myoclonic seizures	n = 12 (2.9%)
Atonic seizures	n = 8 (1.9%)
Mixed seizure types	n = 24 (5.7%)

**Table 4:** Types of Seizures in Adult Patients

TOTAL NUMBER OF ADULT PATIENTS	N = 577
Generalized onset tonic-clonic seizures	n = 411 (71.2%)
Focal aware seizures	n = 38 (6.6%)
Focal impaired awareness seizures	n = 26 (4.5%)
Focal to bilateral tonic-clonic seizures	n = 67 (11.6%)
Absence seizures	n = 7 (1.2%)
Myoclonic seizures	n = 8 (1.4%)
Atonic seizures	n = 2 (0.3%)
Mixed seizure types	n = 18 (3.1%)

EEG was normal in 558 (56%) patients while abnormal EEG was found in 438 (44%) patients. Among abnormal EEGs interictal epileptiform abnormalities alone were found in 73.0% (n = 320), encephalopathy alone in 17.8% (n = 78) and both interictal epileptiform abnormalities and encephalopathy in 9.2% (n = 40) patients (Table 5).

**Table 5:** EEG Patterns

TOTAL NUMBER OF PATIENTS	N = 996
NORMAL EEG	N = 558 (56.0%)
ABNORMAL EEG	N = 438 (44.0%)
Interictal epileptiform abnormalities	n = 320 (32.2% of total, 73.0% of abnormal EEGs)
Encephalopathy pattern	n = 78 (7.8% of total, 17.8% of abnormal EEGs)
Interictal epileptiform abnormalities and encephalopathy pattern	n = 40 (4.0% of total, 9.2% of abnormal EEGs)

## Discussion

The most common type of seizures overall in our study population was generalized onset tonic-clonic seizures which was found in 73% of study population, followed by focal to bilateral tonic-clonic seizures and focal aware seizures. The same three types of seizures dominated the presentation in adult population. The pediatric population presented most often with generalized onset tonic-clonic seizures, followed by mixed seizure types and focal to bilateral tonic-clonic seizures. These findings are similar to many other studies conducted in Pakistan and other countries. Aziz et al. conducted a study according to which the most common category of seizures was generalized onset tonic-clonic seizures followed by focal to bilateral tonic-clonic seizures. The least common category of seizures was absence seizures (1%). The cause of seizures could only be evaluated in 38.4% of their study population. Adequate management of epilepsy was only received by 27% of urban patients and 2% of rural patients [3]. The mean age of Aziz et al.’s study subjects was 13.3 years while the mean age in our study population was 21.4 ± 3.2 years. Aziz et al. conducted another epidemiological study in 1997 comparing the prevalence of epilepsy among population of Pakistan and Turkey. The most common type of seizures was similar among Pakistani and Turkish population [6].

According to previous studies the main cause of seizures in elderly is stroke which accounts for 30-70% of cases. Cerebral tumors cause 10-15% of seizures in this population while metabolic disturbances are responsible for less than 10% seizures. Other causes include head injury, CNS infections and neurodegenerative disorders [7]. In children, the most common causes of seizures include high-grade fever, cerebral palsy, hypoxic-ischemic encephalopathy, infections, intracranial hemorrhage and hydrocephalus [8]. In our study, 58% of seizures were primary while 42% were secondary. This distribution was more even in children with 49.6% primary seizures and 50.4% secondary seizures; the gap widened in adults with 64.3% primary seizures and 35.7% secondary seizures. Among secondary causes, most common overall was neonatal encephalopathy followed by head trauma and CNS infections. Other causes found were cerebral tumors, stroke, metabolic encephalopathy, febrile seizures and cerebral malformations. Again the difference in children and adults was noticeable in causes of secondary seizures. Neonatal encephalopathy, CNS infections and febrile seizures represented the most frequently presenting causes of secondary seizures in children. In adults the top three causes of secondary seizures were head trauma, cerebral tumors and stroke.

Kaur et al. conducted a study on the causes of seizures among adults admitted to a tertiary care setup in India. The most common cause of seizures in their study population was stroke followed by idiopathic causes and CNS infections. Cerebral tumors accounted for 10% of cases while metabolic disturbances were responsible in 12% patients. The most common cause of generalized onset tonic-clonic seizures was idiopathic while the most common cause of focal seizures was stroke. Status epilepticus was most often caused by metabolic derangements. They concluded that advanced age led to reductions in incidence of generalized seizures and focal seizures became more predominant as age progressed [9]. Mac et al. conducted a systemic review on the causes of epilepsy in Asia. Prevalence data from 11 different countries was analyzed. According to this study, head trauma, stroke, CNS infections and trauma during birth account for the most common causes. They concluded that diagnostic and therapeutic modalities were limited in many regions [10].

In our study, the mean age was 21.4 ± 3.2 years. In studies conducted in Pakistan and other underdeveloped countries in Asia, the mean age for epileptic patients lies between 15-20 years. However in developed countries epilepsy has a bimodal distribution. The first peak is after few years of birth whereas the other peak occurs after 65 years of age [11]. Most of our patients were male. Khan et al. conducted a study on the prevalence of epilepsy in children in 2019 in Karachi. He concluded that the prevalence was greater in males compared to females. Epilepsy was present in 22% of his study population with generalized onset tonic-clonic seizure as the most common type [12].

Ullah et al. in 2018 conducted an epidemiologic study on the characteristics of epileptic patients in Khyber Pakhtunkhwa (KPK) province of Pakistan. The mean age of their study population was 18 ± 8 years with majority of patients being male. Most patients from this study belonged to low socio-economic status. The characteristics associated with poor response to management included low socio-economic status, non-compliance to therapy and generalized onset tonic-clonic seizures [4].

Our study found that patients with epilepsy represented 10.5% of the total patient turnover in neurology department of our hospital. Considering the vast number of neurological illnesses this number looks a bit high. This can probably be explained by the fact that internal medicine departments and general practitioners often feel confident in treating some commonly presenting neurological diseases like headache and stroke, but most of the epilepsy patients are referred to neurology services. We also looked into the EEG patterns of our patients and found that 56% of them had normal EEG. 32.2% patients had interictal epileptiform abnormalities, 7.8% had encephalopathy pattern and 4% had both interictal epileptiform abnormalities and encephalopathy pattern. McGinty et al. found that 54.4% of their patients had normal EEG, 24.6% had non-specific abnormalities and 21.1% patients demonstrated interictal epileptiform abnormalities [13].

The limitation of this study is that it represents the patients presenting to a single hospital and therefore generalization over the whole population may not be appropriate. Major strength of this study was a significant sample size spread over two years that minimize the chances of false representation of the actual state of affairs.

Neonatal encephalopathy and head trauma were responsible for secondary epilepsy in majority of pediatric and adult population respectively in our study. Maternal and neonatal health has always been a major concern in Pakistan. This study, by finding neonatal encephalopathy as the leading cause of secondary epilepsy in children, reinforces the need for improving this crucial aspect of society health. Bike riders are frequently seen without helmets on all the major roads of Karachi. Head trauma was consequential for the most number of adult patients in this study; this underpins the need for creating awareness and perhaps implementing more stringent measures to eliminate this harmful habit among the population. Further studies on the causes and types of epilepsy in Pakistan are required as these factors differ between populations and hence the management strategies also differ consequently.

## Conclusions

This study provides an insight into an under-researched area of epilepsy in Pakistan. The causes and types of seizures dictate the best possible treatment options. We found that the most common cause of seizures in our overall study population was primary seizures, though primary and secondary seizures were more evenly present in children. Among secondary causes neonatal encephalopathy stood out as the most common cause in children; head trauma was the predominant cause in adults. Most common type of seizures overall and in adults was generalized onset tonic-clonic seizures, followed by focal to bilateral tonic-clonic and focal aware seizure types. Pediatric patients presented most often with generalized onset tonic-clonic seizures. Mixed seizure types and focal to bilateral tonic-clonic seizures were the next most common type of seizures in children. Further studies are needed in Pakistan so that conclusive data is accumulated in this regard to aid in making better decisions to combat this common disease.
